# A case of *Exophiala dermatitidis* infection in a child after allogeneic stem cell transplantation: case report and literature review of paediatric cases

**DOI:** 10.1099/jmmcr.0.005102

**Published:** 2017-06-26

**Authors:** Dominika Tanuskova, Julia Horakova, Darina Buzassyova, Miroslava Poczova, Ivana Bodova, Peter Svec, Alica Chocholova, Jaroslava Adamcakova, Tomas Sykora, Miroslava Pozdechova, Lucia Geczova, Alexandra Kolenova

**Affiliations:** ^1^​ Department of Paediatric Haematology and Oncology, Haematopoietic Stem Cell Transplantation Unit, Comenius University Children‘s Hospital, Bratislava, Slovakia; ^2^​ Department of Paediatric Anaesthesiology and Intensive Medicine, Comenius University Children‘s Hospital, Bratislava, Slovakia; ^3^​ Department of Mycology, HPL Ltd, The Medirex Group, Bratislava, Slovakia

**Keywords:** *Exophiala dermatitidis*, child, stem cell transplantation, grey sputum, posaconazole

## Abstract

**Introduction.** *Exophiala dermatitidis*is a relatively common environmental black yeast with worldwide distribution and is a rare cause of fungal infection, mostly in patients with certain predisposing factors. Due to the rarity of the infection, little is known about the specific predisposing factors, way of infection or treatment.

**Case presentation.** Here, we report what is to our knowledge the first case of *E. dermatitidis* infection in a child after allogeneic stem cell transplantation. We also review all paediatric cases reported in the literature since 1993.

**Conclusion.** This is, to our knowledge, the first reported case of *E. dermatitidis* infection in a child after allogeneic stem cell transplantation. This report should increase the awareness of *E. dermatitidis* in immunocompromised paediatric patients, particularly after stem cell transplantation.

## Abbreviation

HIV, human immunodeficiency virus.

## Introduction

*Exophiala dermatitidis* is a yeast that can cause a skin, subcutaneous or systemic infection and is a well-known cause of lung infection in patients with cystic fibrosis [1–4]. It is a very rare infection in children. To our knowledge, this is the first case of *E. dermatitis* infection in a child after haematopoietic stem cell transplantation.

## Case report

An 8-year-old boy with secondary acute myeloid leukemia in complete remission after therapy for relapsed medulloblastoma was admitted to Bone Marrow Transplantation Unit in Bratislava for a planned allogeneic stem cell transplantation. The bone marrow transplantation from a 9/10 HLA (human leukocyte antigen) -matched unrelated donor was performed [day (D) 0] after conditioning consisting of treosulfan, cyclophosphamide and melphalan. Anti-thymocyte globulin and cyclosporin A were used for graft-versus-host disease prophylaxis. From D+2, the patient's clinical and laboratory status started rapidly worsening. The patient was subfebrile up to 37.2 °C, tachydyspnoic with insufficient diuresis (oliguria) and fluid retention. The patient was pancytopenic with: high inflammation activity – 158 mg C-reactive protein l^−1^, 1.02 µg procalcitonine l^−1^; nephropathy – 9.9 mmol urea l^−1^, 98 µmol creatinine l^−1^; metabolic acidosis – pH 7.23, pCO_2_3.9 kPa, HCO_3_ 11.9 mmol l^−1^. Due to respiratory and renal failure, the patient was transferred on D+3 to a paediatric intensive care unit. Despite the clinical and laboratory signs of sepsis, the aetiology (the pathogen) was not recognized with repeatedly negative/sterile blood cultures. On D+7, the patient required mechanical ventilation, and from D+9 continuous peritoneal dialysis. On D+9, while positioning him, an asystole occurred and cardiopulmonary resusitation was required. The cardiopulmonary resusitation was successful, but from then onwards continuous adrenalin was needed. On D+13, due to progression of pleural effusion, pleural puncture with a pigtail catheter insertion was performed. Repeatedly tested (cultivation, *Pneumocystis jirovecii* PCR, galactomannan) pleural and peritoneal effusions were negative, as were all routinely monitored swabs, virus PCRs and galactomannan from blood. The patient was treated with wide-spectrum empirical antimicrobial therapy, since no specific pathogen was recognized. After a short period of clinical stabilization, the patient’s clinical and laboratory status started worsening again. He was haemodynamically unstable with progression of multiorgan failure. From D+16, signs of bullous epidermolysis occurred, the skin became dark, even black, on the scrotum, and grey sputum appeared from bronchi aspiration. On D+20, a yeast, *E. dermatitidis*, was identified from several tracheobronchial aspirations and galactomannan was positive from the aspirations – 2.23 (positivity index). The same yeast was then identified from a pleural effusion from D+18, and throat and nose swabs on D+21. The yeast was susceptible to posaconazole, voriconazole and amphotericin B (Table 1). The patient had antimycotic prophylaxis – micafungin from D−13, at a dose of 1 mg kg^−1^, from D+3 the dose was increase to a therapeutic 2 mg kg^−1^, because his condition worsened on empiric broad spectrum antibiotics without any proof of the infection pathogen. Due to kidney failure requiring peritoneal dialysis, treatment with intravenous posaconazole 1×150 mg (the patient was 22 kg at that time) was started. Despite the targeted therapy, the patient died of multiorgan failure on D+25. To the day of his death he did not engraft. Autopsy was not performed. Surprisingly, *E. dermatitidis* was not cultivated from the black lesions of the patient's skin.

**Table 1.** MICs of the *E. dermatitidis* isolate as reported by the Department of Mycology, HPL Ltd, Bratislava, Slovakia

**Antifungal agent**	**Susceptibility, MIC (μg ml^−1^)**
Fluconazole	Semi- susceptible, 3.0
Itraconazole	Susceptible, 0.125
Voriconazole	Susceptible, 0.064
Posaconazole	Susceptible, 0.094
Amphotericin	Susceptible, 0.094
Caspofungin	Resistant, 32
Anidulafungin	Resistant, 32
Micafungin	Resistant, 32

## Discussion

*E. dermatitidis*, previously known as *Wangiella dermatitidis*, belongs to the group of the so-called black yeasts, which is a polyphyletic morphological group within the Ascomycetes that is characterized by melanized cells and yeast-like growth states (multilateral and polar budding cells) in addition to hyphal growth [5]. *Exophiala* spp. are ubiquitous in nature, but are also found in dishwashers, steam-bath facilities and bathrooms [6–9]. They are increasingly being recognized as a cause of human disease resulting in skin, subcutaneous tissue and systemic infections [1–3]. Compared with other *Exophiala* species, *E. dermatitidis*, in particular, appears to be associated frequently with systemic infection, as well as with poorer outcomes [10, 11]. It is well known that *E. dermatitidis* frequently colonizes the airways of patients with cystic fibrosis, can trigger antibody production and may cause significant airway infection in these patients [4]. Matsumoto *et al*. summarized 37 cases of infection published between 1960 and 1992; Suzuki *et al.* summarized 30 cases published between 1993 and 2011, where 80 % of patients had invasive (non-superficial) infections and most of them had predisposing factors (e.g. peritoneal dialysis, leukemia, steroid use, human immunodeficiency virus (HIV) infection, cancer, bronchiectasis and diabetes mellitus) [3, 12]. Chalkias *et al.* in 2014 were the first people who published a case of *E. dermatitidis* infection in an adult patient after allogeneic stem cell transplantation in a setting of severe graft-versus-host-disease [13]. To our knowledge, there has been no case of this infection in a child after allogeneic stem cell transplantation. We have summarized all published paediatric cases of *E. dermatitidis* infection since 1993, including our case, in Table 2. Only nine cases of the infection have been reported in the paediatric population since 1993. Interestingly, only a minority of paediatric patients had predisposing factors in comparison with adults. Our patient presented shortly after allogeneic stem cell transplantation with myeloablative regimen, he did not engraft; therefore, he was pancytopenic and already in a sepsis of unknown origin. The risk of any fungal infection was high. Other predisposing factors are underlying diseases such as cystic fibrosis, acute leukemia and HIV infection.

**Table 2.** Summary of cases of *E. dermatitidis* infection in children without cystic fibrosis since 1993

**No.**	**Age (years)/gender**	**Manifestation**	**Predisposing factor**	**Diagnostic method**	**Treatment**	**Outcome**	**Geographical region**	**Reference**
1	3/M	Fungaemia	Acute leukemia	Culture	Catheter removal, AMPH-B, 5-FC	Survived	Germany	[19]
2	3/M	Fungaemia	HIV infection	Culture	Catheter removal, AMPH-B, ITCZ	Survived	USA	[20]
3	8/M	Systemic	None	Biopsy	AMPH-B, VRCZ	Died	Turkey	[21]
4	3/M	Brain abscess, meningitis	None	Biopsy, culture, PCR	AMPH-B, FLCZ, ITLC	Died	China	[14]
5	11/F	Liver cirrhosis	None	Biopsy, culture, PCR	VRCZ, liver transplantation	Survived	Korea	[22]
6	17/M	Phaeohyphomycosis	None	Biopsy	ITCZ, Op	Survived	Argentina	[23]
7	8/M	Brain abscess	None	Biopsy	AMPH-B, VRCZ, 5-FC	Died	China	[24]
8	16/F	Pneumonia	Cystic fibrosis	Culture	ITCZ, VRCZ	ns	USA	[25]
9	8/M	Pneumonia	AML after BMT	Culture	PSCZ	Died	Slovakia	Our case

5-FC, 5-Fluorocytosine; AML, acute myeloid leukemia; AMPH-B, amphotericin B; BMT, bone marrow transplantation; F, female; FLCZ, fluconazole; ITCZ, itraconazole; M, male; ns, not stated; Op, operation; PSCZ, posaconazole; VRCZ, voriconazole.

Another interesting fact is the neurotropism of *E. dermatitidis* in China – both children reported from China were immunocompetent and had brain abscess, which none of other children had. Involvement of the central nervous system is associated with poor prognosis and both children died. This neurotropism in Asia had been described before, but the explanation remains difficult to understand [14]. Matos *et al.* investigated the molecular diversity of *E. dermatitidis*. On the basis of internal transcribed spacer sequence, fingerprint and small subunit intron data, strains of *E. dermatitidis* were subdivided into groups A and B. All strains from Asia were of the A genotype. Members of group B have been found in Europe and America, but not in Asia [15].

Few treatments were ultimately found to be effective, and the optimal antifungal therapy for these infections is unknown [16]. Data on *in vitro* susceptibility are sparse and seem to poorly correlate with *in vivo* drug efficacy [17]. As it is difficult to treat the disease after the disease has become fulminated, measures should be taken to initiate appropriate therapy as soon as possible [18]. In our case, despite immediate targeted treatment according to the MIC with intravenous posaconazole, the patient died after 5 days of treatment.

The *E. dermatitidis* infection occurred when the patient had already been in a critical state in the paediatric intensive care unit on mechanical ventilation for 17 days. Therefore, it is impossible to know the degree of contribution of the *E. dermatitidis* infection to the death of the patient. However, his state worsened after he acquired *E. dermatitidis* infection and he died shortly after.

The prognosis of any rare fungal infection is always very serious and this type of infection is often a cause of high mortality, 4 out of 9 children with *E. dermatitidis* infection died, which is higher than in adults in the review by Suzuki *et al*., where 4 out of 17 patients died [12].

In conclusion, to our knowledge, this is the first reported case of *E. dermatitidis* infection in a child after allogeneic stem cell transplantation. This report should increase the awareness of *E. dermatitidis* in immunocompromised paediatric patients, particularly after stem cell transplantation.
